# Hemodynamic Assessment Using SPY Laser Fluorescence Imaging During Pancreatoduodenectomy with Common Hepatic Artery Resection

**DOI:** 10.1245/s10434-024-16659-x

**Published:** 2024-12-03

**Authors:** Tomokazu Fuji, Kosei Takagi, Kazuya Yasui, Takeyoshi Nishiyama, Motohiko Yamada, Yasuo Nagai, Noriyuki Kanehira, Toshiyoshi Fujiwara

**Affiliations:** Department of Gastroenterological Surgery, Okayama University Graduate School of Medicine, Dentistry, and Pharmaceutical Sciences, Okayama, Japan

**Keywords:** Pancreatectomy, Pancreatic cancer, Artery resection, indocyanine green, Laser fluorescence imaging

## Abstract

**Background:**

Pancreatectomies combined with arterial resection can be indicated for pancreatic cancer. In a pancreatectomy with arterial resection, intraoperative confirmation of blood flow through reconstructed vessels is crucial. This study highlights the usefulness of SPY laser fluorescence imaging during a pancreatoduodenectomy with common hepatic artery resection (PD-CHAR).

**Patient and Methods:**

A 55-year-old man with borderline resectable pancreatic head cancer underwent a PD-CHAR. After confirming tumor resectability, reconstruction of the CHA to the proper hepatic artery was performed. Subsequently, the superior mesenteric vein was reconstructed.

**Results:**

SPY laser fluorescence imaging demonstrated arterial blood perfusion to the liver through the reconstructed hepatic artery, followed by perfusion from the portal vein. The operation lasted 493 min, with an estimated blood loss of 400 mL. The postoperative course was uneventful with good arterial blood flow.

**Conclusion:**

The SPY Portable Handheld Imager could be valuable for visualizing blood flow in reconstructed vessels and assessing tissue perfusion during a pancreatectomy combined with vascular reconstruction.

**Supplementary Information:**

The online version contains supplementary material available at 10.1245/s10434-024-16659-x.

Pancreatectomy combined with arterial resection is indicated for cancers of the pancreatic neck and body that are in contact with the common hepatic artery.^1^ Recent REDISCOVER guidelines permit careful consideration of arterial resections in highly selected patients who demonstrate involvement of the hepatic artery but not of the superior mesenteric artery, in high-volume centers.^2,3^ In procedures involving a pancreatectomy with arterial resection, intraoperative confirmation of blood flow through reconstructed vessels is essential^1^ Herein, we demonstrate the usefulness of SPY laser fluorescence imaging during a pancreatoduodenectomy with common hepatic artery resection (PD-CHAR) (Supplementary Video 1).

## Case

**Fig. 1:**
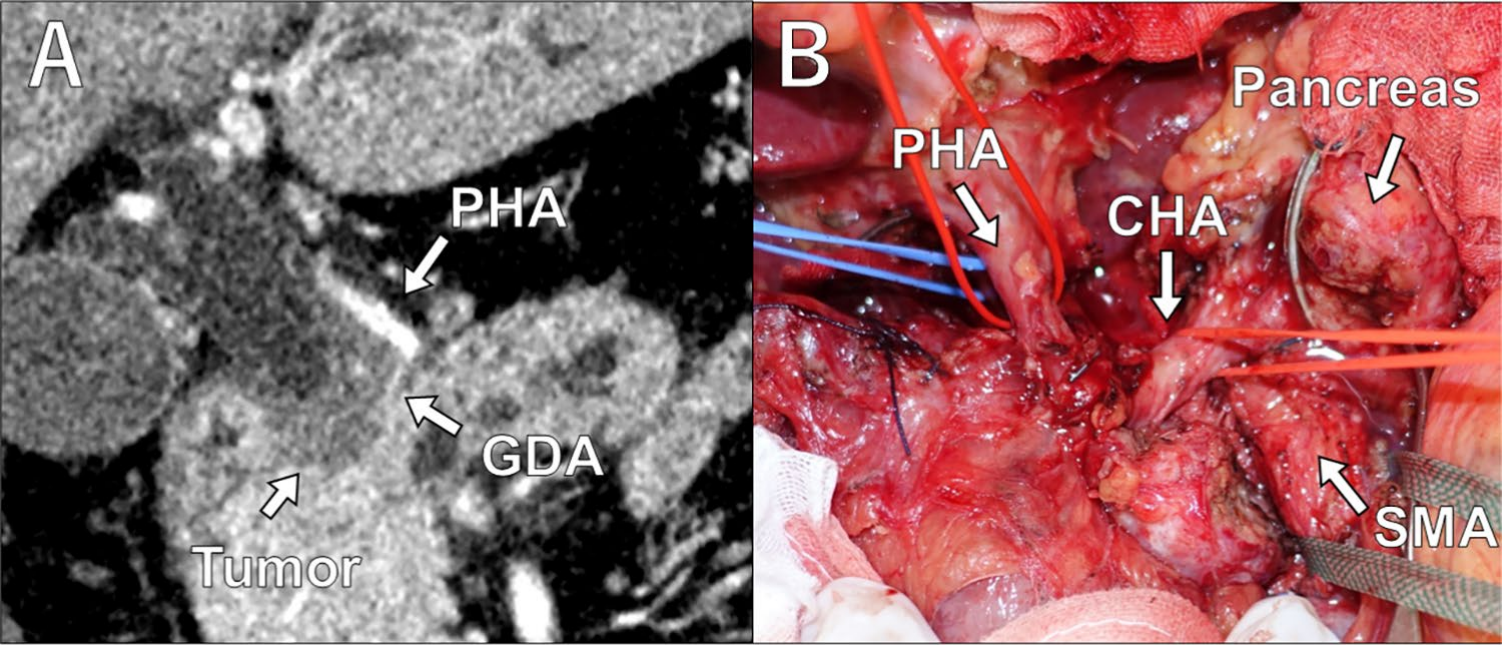
**A** Preoperative computed tomography images demonstrative of pancreatic head cancer in contact with the common hepatic artery (CHA), gastroduodenal artery (GDA), and proper hepatic artery (PHA). **B** Intraoperative findings indicate arterial involvement to the CHA, GDA, and PHA

A 55-year-old man was diagnosed with borderline resectable pancreatic head cancer, exhibiting arterial involvement to the common hepatic artery (CHA), gastroduodenal artery (GDA), and proper hepatic artery (PHA) (Fig. 1A). Following 4 months of systemic chemotherapy with gemcitabine and nab-paclitaxel, the patient underwent a PD-CHAR as a conversion surgery. The response evaluation of systemic chemotherapy indicated a partial response.

## Surgical Technique

Initially, tumor resectability was confirmed through staging laparoscopy, which revealed no liver metastasis or peritoneal dissemination. Following the division of the gastrohepatic ligament, the hepatoduodenal ligament was dissected to encircle the PHA and portal vein (PV). The roots of the CHA and splenic artery (SA) were carefully dissected and encircled to confirm the tumor-free distance. Subsequently, the pancreatic body was dissected and transected at the superior mesenteric artery (SMA), where a negative margin was confirmed through frozen section analysis. Systematic mesopancreas dissection was performed, incorporating nerve-sparing dissection techniques for the SMA (level 2) and the celiac axis.^4^ At this point, the specimen was connected only with the hepatic artery and PV (Fig. 1B).

Arterial resection and reconstruction were performed first. The CHA and PHA were clamped and transected. Reconstruction of the CHA was achieved through a running end-to-end anastomosis with the PHA using a 6-0 Prolene suture. Subsequently, the superior mesenteric vein and PV were clamped and divided, followed by the resection of the specimen. The PV was then reconstructed through a running end-to-end anastomosis.

**Fig. 2:**
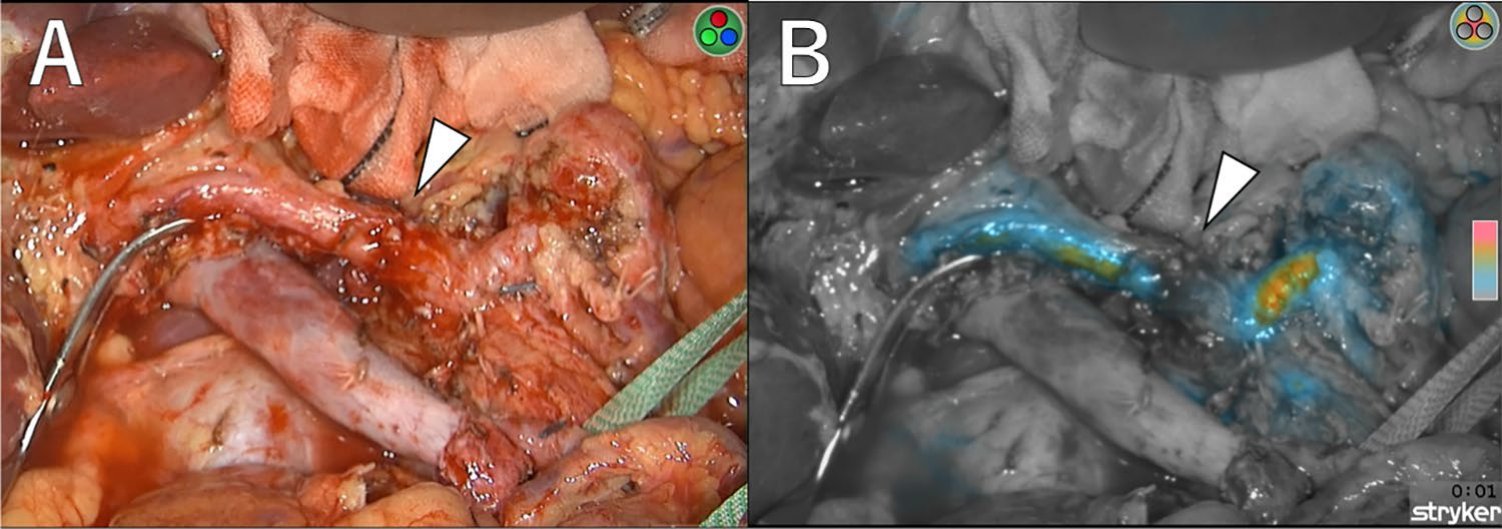
**A** The SPY intraoperative perfusion assessment system was utilized during the pancreatectomy. The CHA and PHA were anastomosed in an end-to-end fashion (triangle). **B** In color-segmented fluorescence mode, arterial blood perfusion was immediately visualized after the injection of indocyanine green

After reconstruction of blood vessels, the SPY Portable Handheld Imager (SPY-PHI, Stryker, USA) was used to confirm blood supply in real time on the monitor (Fig. 2A). Following an injection of indocyanine green (ICG) (2.5 mg/body), arterial blood perfusion to the liver via the reconstructed hepatic artery was immediately visualized, followed by perfusion of the PV (Fig. 2B). The entire liver blood flow was observed in the overlay fluorescence mode.

The operation lasted 493 min, with an estimated blood loss of 400 mL. The postoperative course was uneventful with good arterial blood flow confirmed using computed tomography.

## Discussion

This study presented the usefulness of SPY laser fluorescence imaging in a PD-CHAR. Although Doppler ultrasound remains a standard tool to evaluate blood flow in reconstructed vessels, the SPY intraoperative perfusion assessment system has been proposed as a valuable option for perioperative visualization of tissue perfusion.^5^ In the present case, the SPY-PHI system facilitated real-time perfusion assessment of reconstructed vessels and related tissue perfusion using near-infrared fluorescence imaging during a PD-CHAR. Following an injection of ICG, a fluorescence signal was immediately detected from the CHA to the PHA, extending to the hilar plate, and subsequently indicating PV flow throughout the liver. A previous study reported the efficacy of intraoperative ICG fluorescence imaging for blood flow evaluation of reconstructed vessels during PD-CHAR.^1^ However, to the best of our knowledge, this study represents the first clinical experience utilizing the SPY-PHI system as an evaluation tool for reconstructed vessels in PD-CHAR, where clinical judgment may be challenging.

In the present case, the splenic vein was divided and not reconstructed. The division of the splenic vein allowed easier dissection around the SMA and celiac axis. Moreover, complete detachment of the pancreatic head was achieved with the specimen remaining connected only with the hepatic artery and PV, thereby facilitating arterial reconstruction. In contrast, special caution was necessary regarding the left-sided portal hypertension following the division of the splenic vein.^6^ Furthermore, an interposition graft is often necessary to reconstruct the splenic vein. Therefore, it is important to evaluate the advantages and disadvantages of its reconstruction.

As per the REDISCOVER guidelines, arterial resections during pancreatectomy should be performed by skilled pancreatic surgeons in centers of excellence.^2,3^ A recent single-center study on pancreatectomy with arterial resection by Napoli et al. reported feasible and improved postoperative outcomes despite a steep learning curve.^7^

In conclusion, the SPY-PHI system could be useful for visualizing blood flow in reconstructed vessels and for assessing tissue perfusion during pancreatectomies combined with vascular reconstruction.

## Supplementary Information

Below is the link to the electronic supplementary material.

Supplementary file1 (MP4 199251 kb)
